# Clinical and Radiological Spectrum of Posterior Reversible Encephalopathy Syndrome: Does Age Make a Difference? – A Retrospective Comparison between Adult and Pediatric Patients

**DOI:** 10.1371/journal.pone.0115073

**Published:** 2014-12-16

**Authors:** Eberhard Siebert, Georg Bohner, Matthias Endres, Thomas G. Liman

**Affiliations:** 1 Department of Neuroradiology, Charité - Universitätsmedizin Berlin, Berlin, Germany; 2 Department of Neurology, Charité - Universitätsmedizin Berlin, Berlin, Germany; 3 Center for Stroke Research Berlin, Charité - Universitätsmedizin Berlin, Berlin, Germany; 4 German Center for Cardiovascular Research (DZHK) Partner Site, Charité - Universitätsmedizin Berlin, Berlin, Germany; 5 German Center for Neurodegenerative Diseases (DZNE) Partner Site, Charité - Universitätsmedizin Berlin, Berlin, Germany; Maastricht University, The Netherlands

## Abstract

**Background:**

Posterior reversible encephalopathy syndrome (PRES) is a serious and increasingly recognized disorder, but data from observational studies on clinicoradiological differences between etiologies and age groups are limited. In this study, we aimed to investigate the clinical and imaging characteristics of PRES in children compared to adults in a large cohort.

**Methods:**

We retrospectively reviewed the radiological report data bases between January 1999 and August 2012 for patients with PRES (total of 110 patients). Patients fulfilling the criteria for PRES after detailed investigation of clinical charts and imaging studies were separated into children (<18years) and adults (≥18years). Various imaging features at onset of symptoms and on follow-up as well as clinical and paraclinical data were analyzed.

**Results:**

A total of 19 pediatric and 91 adult patients with PRES were included into the study. In pediatric PRES patients, seizures were significantly more frequent as initial PRES-related symptom (p = 0.01). In addition, in children the superior frontal sulcus topographic lesion pattern occurred as frequent as the parieto-occipital one and was significantly more prevalent than in adults (p = 0.02). In contrast, in adults visual disturbances tended to occur more frequently than in children (p = 0.05). Also, severity of edema tended to be greater in adults than in children (p = 0.07).

**Conclusion:**

In our PRES cohort, we found relevant clinicoradiological differences between pediatric and adult PRES patients. However, prospective studies are warranted to establish factors that are specifically associated with pediatric PRES.

## Introduction

Posterior reversible encephalopathy syndrome (PRES) as defined by Hinchey and colleagues in 1996 describes a phenomenon of transient cerebral vasogenic edema occurring preferentially in the posterior circulation [1]. Clinically, PRES is characterized by headaches, seizures, reduced consciousness, visual and other focal neurological symptoms [2]–[3]. There is a long and growing list of predisposing factors and diseases, including various cytostatic and immunosuppressive drugs in hemato-oncologic and autoimmune diseases, solid and bone marrow transplantation, hypertension, toxemia of pregnancy, connective tissue diseases and renal diseases [4]–[5]. The radiological spectrum of PRES has expanded in the recent past comprising variants with various distribution patterns of brain edema, development of cytotoxic edema, infarctions, hemorrhages, laminar necroses and glioses [3], [6]–[10]. Clinical symptoms are typically transient and include seizures, headaches, visual deficits, focal neurological symptoms as well as various degrees of reduced consciousness [2], [5]. The putative pathophysiological principle is impaired cerebrovascular autoregulation combined with endothelial dysfunction causing reversible vasogenic edema of the brain [4].

Whereas many papers have studied the clinical and imaging characteristics of PRES in the adult population and some papers have focused on the pediatric age group, there is no radiological or clinical comparison of PRES between the adult and the pediatric population in the literature to the best of our knowledge [2], [3], [4], [6]–[13]. Since PRES-predisposing disorders vary for different age groups and the pediatric brain differs in many aspects from the adult one including differences in susceptibility to a wide variety of noxious substances, differences in cerebral hemodynamics and vasoregulation as well as a higher regeneration potential, the radiological picture as well as the clinical course of this disease might differ [12], [13], [16]–[20]. Therefore, we compared a wide range of clinical as well as radiological items of the adult cohort of the retrospective Berlin PRES study conducted in all three university hospitals of Berlin and our pediatric PRES collective [3], [14], [21]–[22].

## Methods

### Patients

Inclusion criteria and selection and of patients into the retrospective Berlin PRES study were described previously [3], [14], [21]–[22]. In summary, a radiological report data base search of the authors' university hospitals was performed for the following items cited on MRI reports between January 1999 and March 2011: posterior reversible leucoencephalopathy, PRES, posterior reversible encephalopathy, hypertensive encephalopathy, toxemia of pregnancy, preeclampsia, eclampsia, tacrolimus, neurotoxicity, FK-506, cyclosporine, transplantation, systemic sclerosis, systemic lupus erythematodes, Wegener's granulomatosis and scleroderma.

Medical report and brain imaging data of identified candidates were analyzed for a) radiological findings compatible with PRES, i.e. variable degrees of vasogenic edema, variable degrees of reversibility and b) a clinical constellation compatible with PRES. If both were present, patients were included into the study.

### Clinical Evaluation

All available clinical records were screened for data known or suspected to be related to the development of PRES (see above listed search items). Demographic data, neurological symptoms at initial clinical presentation of PRES, related parameters of predisposing diseases, laboratory data as well as blood pressure levels at time of onset of PRES-related symptoms were acquired. Etiology of PRES was categorized in autoimmune disorders (e.g. systemic lupus erythematodes), immunosuppression (e.g. solid organ transplantation), chemotherapy and other/unknown (e.g. evident toxic associations, sepsis) as done previously [3], [14], [21]–[22]. In cases with more than one clinical association, the clinically dominant feature was used for analyses and tabulation. Furthermore, laboratory parameters i.e. coagulation parameters (International Normalized Ratio (INR), prothrombin time (PT), partial thromboplastin time (PTT)), thromobocytes, creatinine and C- reactive protein (CRP) were acquired from available clinical records within a maximum range of 3 days from initial onset of PRES symptoms.

Arterial hypertension was defined as mild (i.e., systolic blood pressure 140–159 mmHG), moderate (160–179 mmHG), and severe (>180 mmHG) for adults. For children systolic or diastolic blood pressure values above the 95 percentile for the appropriate age group were defined as arterial hypertension [23]. Coagulation state was categorized as 1) normal or subtherapeutic coagulation or 2) altered coagulation state including therapeutic, oral or intravenous medication i.e. heparin, phenprocoumon/Vit-K antagonists that may affect coagulation cascade or/and laboratory findings of elevated INR, PTT, PT and/or thrombocytopenia. Cut-off values used in our analysis for highly elevated C-reactive protein were 10 mg/dl and 0.9 mg/dl for creatinine. Length of hospital stay and discharge status (home, inpatient rehabilitation/other hospital, and in-hospital death) were acquired for all PRES patients included in the study.

### Imaging Evaluation

Imaging evaluation methods were utilized as described in detail in previous publications on the Berlin PRES study [3], [14], [21]–[22]. In short, MR imaging was performed in all patients. All studies included axial T1- and T2 weighted images. Axial Fluid-attenuated inversion-recovery (FLAIR) images, diffusion-weighted imaging sequences (DWI) including ADC (apparent diffusion coefficient) map, T2* weighted sequences and contrast-enhanced T1 weighted sequences were available in 98, 80, 76 and 81 patients, respectively. Scans were assessed for extent of edema (mild: limited cortex and white matter edema with or without limited deep white matter extension; moderate: white matter and cortex edema with limited ventricle surface extension; and severe: diffuse, widely confluent white matter and cortex edema with extensive ventricle contact or ventricle deformity) and hemorrhage (parenchymal hematoma, microbleeds, subarachnoid blood). Diffusivity of edematous lesions was characterized as purely vasogenic or as presence of cytotoxic edema components within the edematous areas (with foci of 1. ADC reduction, or 2. ADC pseudonormalisation within vasogenic edema regarded as cytotoxic edema components).

### Follow-up imaging

If available, follow-up imaging was evaluated for restitution of edema and occurrence of residual structural lesions as described previously [16]. Regression of edema was graded as complete, incomplete, constant or progressive edema. Residual structural lesions were divided into none, small white matter gliosis, post hemorrhagic residua, infarction, atrophy and cortical laminar necrosis.

### Ethics

The retrospective Berlin PRES study was approved by the institutional ethics committee of Charité Universitaetsmedizin-Berlin. All clinical investigation has been conducted according to the principles expressed in the Declaration of Helsinki. Patient records and information were anonymized and de-identified prior to analysis.

### Statistical Analysis

The Mann-Whitney test was used to test differences in continuous variables, and the χ2 test for those in proportions. All statistical test were 2-tailed, and statistical significance was set at p<0.05. SPSS software (SPSS 18, Chicago) was used for all analyses.

## Results

Between January 1999 and August 2012 110 PRES cases were identified on the basis of the above described clinicoradiological criteria and were included into the study. All patients had complete available medical data records on demographic variables, clinical (symptoms and blood pressure values at onset of PRES) and laboratory data (creatinine, C-reactive protein, coagulation status), brain MR imaging as well as duration of hospital stay except of 3 patients whose exact blood pressure levels at initial PRES toxicity were unknown.

Nineteen patients were children <18 years of age (17.3%) while 91 patients were adults (82.7%). Median age was 9 (IQR 7–12) and 48 (IQR 29–64), respectively. Clinical and radiological characteristics are shown in tables 1 and 2.

**Table 1:** Description of pediatric versus adult patients with PRES.

	All	Pediatric patients	Adult patients	*p-value*
**n**	**110**	**19**	**91**	*-*
				
**Age in years, median (IQR)**	**36 (22–61)**	**9 (7–12)**	**48 (29–64)**	*-*
				
**Female sex, n (%)**	**75 (68.1%)**	**7 (36.8%)**	**68 (74.7%)**	*-*
				
Systolic BP in mmHG, median (IQR)*	**160 (140–180)**	**140 (124–160)**	**170 (150–180)**	*0.001*
				
**Age adjusted Arterial Hypertension, n (%)**	**92 (83.6%)**	**15 (78.9%)**	**77 (84.6%)**	*0.4*
**Etiology/Toxic Association, n (%)**				*<0.001*
**Immunosuppression**	**25 (22.7%)**	**3 (15.8%)**	**22 (24.2%)**	
**BMT**	**5 (4.5%)**	**1 (5.3%)**	**4 (0.4%)**	
**SOD**	**20 (18.2%)**	**2 (10.6%)**	**18 (19.8%)**	
**Autoimmune Disorders**	**22 (20.0%)**	**11 (57.9%)**	**11 (12.1%)**	
**Chemotherapy**	**21 (19.1%)**	**4 (21.1%)**	**17 (18.7%)**	
**Pre-/Eclampsia**	**25 (22.7%)**	**0**	**25 (27.5%)**	
**Infection/sepsis**	**6 (5.5%)**	**0**	**6 (6.6%)**	
**Other/Unknown**	**11 (10.0%)**	**1 (5.3%)**	**10 (11.0%)**	
**Initial Neurological Symptoms, n (%)**				
**Seizures**	**78 (70.9%)**	**18 (94.7%)**	**60 (65.9%)**	*0.01*
**Visual Disturbances**	**34 (30.9%)**	**2 (10.5%)**	**32 (35.0%)**	*0.05*
**Altered Mental State**	**42 (38.2%)**	**5 (26.3%)**	**37 (40.7%)**	*0.3*
**Paresis**	**16 (14.5%)**	**4 (21.1%)**	**12 (13.2%)**	*0.47*
**C-reactive Protein in mg/dl, mean ± SD**	**4.2±6.4**	**1.7±2.4**	**4.8±6.8**	*0.01*
**Creatinine in mg/dl, mean ± SD**	**1.8±1.9**	**1.4±2.0**	**1.7±1.9**	*0.5*
				
**Length of Hospital Stay in Days, median (IQR)**	**41.2±40.6**	**50±52**	**39±37**	*0.2*

Exact RR-levels at initial PRES toxicity missing in 3 patients (1 pediatric, 2 adult).

**Table 2:** Radiological features in pediatric versus adult patients with PRES.

Radiological features	All	Pediatric patients	Adult patients	*p-value*
n (%)	110	19	91	
**Edema Grading**				*0.07*
mild	61 (55.5%)	15 (78.9%)	46 (50.5%)	
moderate	30 (27.3%)	2 (10.5%)	28(30.6%)	
severe	19 (17.3%)	2 (10.5%)	17 (18.7%)	
**Pattern**				*0.02*
Parieto-occipital	59 (53.6%)	8 (42.1%)	51 (56.0%)	
Superior frontal sulcus	18 (16.4%)	7 (36.8%)	11 (12.1%)	
Central	14 (12.7%)	4 (21.1%)	14 (15.4%)	
Holohemispheric watershed	19 (17.3%)	0	15 (16.5%)	
**Hemorrhage**				*0.08*
None	78 (70.9%)	12 (63.2%)	66 (72.55)	
Minute	14 (12.7%)	2 (10.5%)	12 (13.2%)	
Sulcal subarachnoidal	5 (4.5%)	0	5 (5.5%)	
Parenchymal	12 (10.9%)	4 (21.1%)	8 (8.8%)	
**Cytotoxic edema components^1^**				*0.08*
Yes	27 (33.8%)	4 (40%)	23 (32.9%)	
No	53 (66.3%)	6 (60%)	47 (67.1%)	
**Enhancement^2^**	50 (61.7%)	8 (50%)	42 (64.7%)	*0.45*
No enhancement Leptomeningeal	11 (14.8%)	3 (18.8%)	8 (12.3%)	
Parenchymal	20 (24.7%)	5 (31.2%)	15 (23.1%)	
**Edema restitution** 3				*0.3*
Complete resolution	37 (57.8%)	11 (78.6%)	26 (52%)	
Incomplete resolution	16 (25%)	2 (14.3%)	14 (28%)	
Progress	8 (12.5%)	1 (7.1%)	7 (14%)	
Constancy	3 (4.9%)	0	3 (6%)	
**Structural residua** 3				*0.5*
Total	64	14	50	
No residue	35 (54.7%)	8 (57.1%)	27 (54.4%)	
Gliosis	8 (12.5%)	0	8 (16.0%)	
Infarction	2 (3.1%)	1 (7.1%)	1(2.0%)	
Hemorrhagic residua	5 (7.8%)	2 (14.3%	3 (6.0%)	
Cerebral atrophy	1 (1.6%)	0	1 (2.0%)	
Laminar necrosis	13 (20.3%)	3 (21.4%)	10 (20.0%)	

1 Diffusion weighted imaging was performed in 80 patients (10 pediatric, 70 adults).

2 Contrast-enhanced imaging was performed in 81 patients (16 pediatric, 65 adults).

3 Follow-up imaging was performed in 64 patients (14 pediatric, 50 adults).

In univariate analyses, significant differences regarding clinical and paraclinical variables were found between pediatric and adult patients for seizures as presenting neurological symptom (*p = 0.01*), absolute blood pressure levels (*p = 0.01*), etiology of PRES (*p<0.001*) and CRP values *(p = 0.01)*. Furthermore, there was a borderline significance for visual disturbances occurring more frequently in adults than in children (*p = 0.05*). Significant differences concerning imaging findings were found for topographic lesion distribution patterns *(p = 0.02)* only, with the superior frontal sulcus pattern being more frequent in children. There was a trend towards less severe edema grades in children as opposed to adults that did not reach statistical significance *(p = 0.07)*.

No differences were found for creatinine values, length of hospital stay as well as all other radiologic characteristics (hemorrhage, cytotoxic edema, pathologic contrast enhancement, edema restitution, and structural residua).

## Discussion

This study is the first to compare the adult and the pediatric PRES population systematically, to the best of our knowledge. In 1996 Hinchey and colleagues introduced the posterior reversible encephalopathy syndrome (PRES) as a specific clinico-radiological disease entity defined by a predominantly vasogenic and potentially reversible edematization of the brain with preferential involvement of the posterior circulation territories [1]. Since then the syndrome has been recognized increasingly both in adults as well as in children [1]–[3], [6], [14], [24]–[26].

Our study shows that clinico-radiological differences between pediatric and adult PRES patients do exist. Seizures were significantly more frequent in pediatric PRES patients as initial PRES-related symptom than in adults. Visual disturbances tended to be more frequent in adults. Furthermore, in our cohort the superior frontal sulcus topographic lesion pattern occurred as frequently as the parieto-occipital one in children and was significantly more prevalent in children than in adults. Severity of edema tended to be greater in adults than in children. Blood pressure levels at onset of toxicity were higher in adults. However, after adjustment for age the frequency of arterial hypertension in both age groups did not differ.

Our study underlines that both pediatric and adult PRES are heterogeneous entities comprising a variety of clinical and imaging features which is in line with the literature [1]–[3], [6], [14], [24]–[26]. Accordingly, PRES in both age groups occurred in a broad range of disorders and predisposing conditions in our study. In both age groups the range of PRES-associated diseases was similar, with the main differences between both cohorts being (pr)eclampsia manifesting only in adults, and children having significantly more autoimmune predisposing diseases, mainly those with renal insufficiency such as glomerulonephritis treated with immunosuppressants which are highly prevalent in this age group. Furthermore case-mix phenomena specific to our institutions might have contributed to this differential distribution. In both age groups PRES occurred frequently in hemato-oncologic patients associated with cytostatic drugs. It has been hypothesized that endotheliotoxic effects of both immunosuppressive as well as cytostatic drugs may lead to blood-brain barrier disturbances as well as impairment of cerebral vascular autoregulation, especially if in combination with other predisposing factors such as hypertension, autoinflammatory states, and endogenic or exogenic metabolic - toxic substances [2], [4], [13]–[15], [26].

Clinically, the most striking aspect was that seizures were significantly more frequent in the pediatric age group as compared to the adult age group. Accordingly, other authors reported high frequencies of seizures in pediatric PRES and a recent pediatric study showed that in children with PRES secondary to renal disease the prevalence of seizures was greater in younger children as compared to older children [15], [27]. Indeed experimental data suggest that exposure to calcineurine inhibitors causes more severe neurotoxicity at a young age due to an increased permeability of the immature blood-brain barrier allowing PRES - mediating circulating substances to act on the brain [4], [5], [20]. These factors might contribute to the high incidence of seizures observed in pediatric PRES, possibly sharing pathophysiological similarities with pediatric febrile seizures, a common pediatric condition, that might also be partially triggered by fever- associated circulating proinflammatory cytokines [28].

Another finding in our study was that visual disturbances tended to be less frequent in the pediatric population compared with adults. This might be explicable by our observation that the superior frontal sulcus pattern was significantly more frequent and the typical parieto-occipital pattern was less frequent in children with a relative sparing of the optic radiations and the occipital visual cortices as opposed to adults [3], [6], [14]. Since the superior frontal sulcus corresponds to the anterior watershed region, the high prevalence of the superior sulcus pattern in our study might indicate that in pediatric PRES hemodynamic instability caused by cerebrovascular dysregulation might be an important pathophysiological element. Indeed, it has been hypothesised that a failure of autovasoregulation with transient overshooting vasoconstriction and subsequent reactive vasodilation might be an explanation for this predominant anterior watershed distribution of edematous lesions seen in the pediatric age group with putative endotheliopathy and resulting blood - brain barrier disruption as aggravating factors [14], [16], [29]. Correspondingly in children the cerebrovascular autoregulatory blood pressure range in which cerebral blood flow is maintained constant is narrower and lower than in adults possibly leading to a greater vulnerability of the anterior watershed zone [16], [18]. Both autovasoregulatory response and susceptibility to hypoperfusion secondary to reactive vasoconstriction were shown to improve with increasing maturity of the involved brain region and thus age [18]. Hence, it has been suggested that children might be more prone to PRES as well as its complications [30]. This study did not investigate possible effects of arteriolosclerosis in advanced age on the course of PRES. However, in two recent PRES studies, age was not significantly associated with outcome [21], [31]. One might wonder whether arteriolosclerosis in older patients might add to impaired vasoregulation and thus aggravate the course of PRES. Interestingly, it is known, that in the presence of chronic hypertension, which is has a high prevalence in older individuals and is one of the main risk factors for arteriolosclerosis, cerebral autovasoregulation can shift to higher values [4], [32]. Considering the hyperperfusion theory of PRES, an up-shifted autoregulation might even act protectively against hyperperfusion whereas considering the hypoperfusion theory of PRES a less reactive autoregulation might reduce vasospasm and subsequent ischemia and reactive edema [4]. Further studies are needed to adequately address this point.

Although PRES is generally completely reversible, persistent lesions are well recognized in adults and children [2], [3], [5], [6], [8], [11], [13], [14], [27]. In this study we also compared the frequency of complications as well as residual structural lesions between the pediatric and the adult age groups. For PRES - related complications such as hemorrhage and cytotoxic edema, we did not find any significant differences between the pediatric and adult age groups. Furthermore, no significant differences for restitution of edema as well as resulting structural residua, such as laminar necroses, infarctions, glioses, post hemorrhagic residua and atrophy, were noted.

The seemingly low rate of complete resolution of edematous lesions (57.8%) is well in line with the literature and is most likely due to the following confounding facts and should not mislead one to call the general reversibility of PRES into question [2], [13], [31]: The timing of the follow up imaging varied widely in our series and likely in most other retrospective series in the literature. In many cases patients did not get neuroimaging after complete resolution of their symptoms. These cases do not appear in follow-up subgroup analyses. On the contrary, one reason to perform a follow-up MRI is the incomplete recovery of a patient, who will likely show an incomplete resolution of imaging changes. Both factors bias such analyses. Furthermore, residual edematous changes are known to persist for longer than the clinical symptoms, usually a couple of days to several weeks. All these factors likely contribute to these relatively high rates of incomplete neuroimaging resolution in this and other studies.

Foci of cytotoxic edema were present in 33.8% of cases in our series which is at the upper end of published values [7], [10]. However in children a very similar rate of cytotoxic edema (36%) was reported [25]. A possible explanation for the relatively frequent occurrence of cytotoxic foci is that we determined these as introduced by Covarrubias who had with 27% of cytotoxic lesions a similar frequency as we did [7]. According to this method areas of vasogenic edema with pseudonormalised ADC contain components of cytotoxic edema, whereas in other studies only areas of reduced ADC were regarded as cytotoxic edema [6], [10], [33]. Nearly all of our cases with cytotoxic edema components consisted of small punctuate or short cortical gyriform foci, not of territorial infarctions, similar to reported by McKinney [10]. Accordingly, in two recent studies ischemic complications or cytotoxic edema in PRES were not significantly associated with outcome [21], [31].

The pathophysiology of PRES as well as the occurrence of cytotoxic foci in PRES is still not completely understood and there are two opposing theories which are discussed controversially in the literature with theory 1 being more popular at present [4]: 1. Hypertension with consecutive failed autoregulation and hyperperfusion. 2. Vasoconstriction with consecutive hypoperfusion and ischemia.

In theory 1 severe hyperperfusion might lead to an increase in local pressure impairing microcirculation and thus leading to ischemia [7], [10]. In theory 2, which is supported by the frequent imaging finding of transient multifocal vessel stenoses in PRES the vasoconstriction might directly induce ischemia [4], [10], [34]. Age dependent differences in cerebral autovasoregulation and cellular susceptibility to ischemia might modulate these processes in both theories.

We acknowledge the following limitations of this study. Since inclusion into the study was based on a search of radiology reports, cases may have been missed. Furthermore, the study cohort is likely subject to a hospital-specific case mix and its findings might not be universally transferable. Etiology and toxic association were categorized according to the predominating clinical feature, not reflecting the fact that in some cases multiple associations were present. Furthermore, timing and specifications of the MR investigations were non uniform both in the acute phase and during follow-up. Finally, general limitations inherent to retrospective studies also apply to this work.

To conclude, we describe both clinical and radiological differences between pediatric and adult PRES. Seizures were more frequent in pediatric PRES than in adults. Visual disturbances tended to be more frequent in adults. In children the superior frontal sulcus topographic lesion pattern was more prevalent than in adults.
